# Supplementary material for: Patterns of Intron Gain and Loss in Fungi
Source: PLoS Biol. 2004 Nov 30;2(12):e422. doi: 10.1371/journal.pbio.0020422 (PMC532390; doi:10.1371/journal.pbio.0020422)
Supplement: Table S1 — Also available at http://genes.mit.edu/NielsenEtAl/. (4.3 MB ZIP). [file pbio.0020422.st001.zip › NielsenEtAl/html/277.html]

AN2243.1.NCU07732.1.MG01743.1.FG09554.1


```
 CLUSTAL W (1.82) Multiple Sequence Alignments - Introns Inserted


Sequence 1: NCU07732.1	453 aa
Sequence 2: FG09554.1	456 aa
Sequence 3: MG01743.1	471 aa
Sequence 4: AN2243.1	417 aa
Alignment Length: 486 aa
Number Identitical Residues: 257 aa
Alignment Score (without introns) 12186


MG01743.1 	MMLSRVSFRMAAATKTAPAFRAAGALASNARPAAKQLRFVSSSQQQQFAHGSKGRKMPAQ
NCU07732.1	-MFSRLAARLP---KASALN----------GVAARQVRNLS----QPAITGSKGRNMPAR
FG09554.1 	-MFSRLASFANPAAKSASAS----------GRTMLQVRYLS----EKAVAGSKGRAMPFS
AN2243.1  	MMFSRFFKAVP----------------------ARAPAFSS------PLPVYQARTMAT-
          	 *:**.                                  *           :.* *.  

MG01743.1 	RSRATAPVAGLDATFTIR0DGPVFHGTAYGANSNISGEAVFTTSLVGYPESMTDPSYRGQ
NCU07732.1	EPRTTAAATGAEATFTIR0DGPVFQGTAFGANTNISGEAVFTTSLVGYPESMTDPSYRGQ
FG09554.1 	AVRATAPAASLPATLTIR0DGPVFQGKAFGANANISGEAVFTTSLVGYPESMTDPSYRGQ
AN2243.1  	-VRNQRPVER--ATFTIR~DGPIFHGKSFGARTTISGEAVFTTSLVGYPESLTDPSYRGQ
          	  *   ..    **:*** ***:*:*.::**.:.*****************:********

MG01743.1 	ILVFTQPLIGNYGVPSKERDEYNLLKYFESPHIQCAGVVVSDIAEKYSHWTAVEGLNEWC
NCU07732.1	ILVFTQPLIGNYGVPSNERDEFNLLKYFESPHIQCAGIVVSDVATQYSHWTAVQSLGEWC
FG09554.1 	ILVFTQPLIGNYGVPSAARDEFNLLKYFESPHVQCAGIVVSDVAVNYSHWTAVESLGEWC
AN2243.1  	ILVFTQPLIGNYGVPSTERDRHGLLKYFESPNLQAAGVVVADVAEQYSHWTAVQSLGEWC
          	****************  **...********::*.**:**:*:* :*******:.*.***

MG01743.1 	AREGVPIISGVDTRAIVTHLREQGSSLARITIGQEYDADEDESFVDPGLINLVKRVSTKA
NCU07732.1	ASEGIPAISGVDTRAIVTYLREQGSSLARISIGDEYDADEDEGFIDPGQINLVKRVSTKA
FG09554.1 	AREGVPAISGVDTRAIVTHLREQGSSLARISIGEEYDADEDEGFVDPGQINLVKRVTTKA
AN2243.1  	AREGVPAISGVDTRAIVTYLREQGSSLARITVGEEYDADQDEAFTDPEQIHLVRQVSTKA
          	* **:* ***********:***********::*:*****:**.* **  *:**::*:***

MG01743.1 	PFVVESPDPTFHIGLIDCGVKENILRSLVSRGASVTVFPYDYPIHKVASNFDGVFISNGP
NCU07732.1	PFVVTNPNAKFHVALIDCGVKENILRSLVSRGASVTVFPYNYPIHKVAENFDGVFISNGP
FG09554.1 	PFVVESPGASLHVALIDCGVKENILRSLVSRGASVTVFPYDYPIHKVSQHFDGVFISNGP
AN2243.1  	PFHVSAADPQCHVAVLDCGVKENILRSLVSRGASITVFPFDYPIHKVAHHFDGVFISNGP
          	** *  ...  *:.::******************:****::******: :**********

MG01743.1 	GDPTHCQETIYNLRRLMETSPVPIMGICLGHQLLALAVGARTLKLKYGNRAHNIPALD0L
NCU07732.1	GDPTHCQETVYNLAKLMETSPIPIMGICLGHQLLALAVGAKTIKLKYGNRAHNIPALD~L
FG09554.1 	GDPTHCQATVHNLARLMETSNIPIMGICLGHQLLALAVGARTIKLKYGNRAHNIPALD~L
AN2243.1  	GDPTHCQDTTYHLRRLMETSQVPIFGICLGHQLLALAA--------------------~-
          	******* * ::* :***** :**:************.                      

MG01743.1 	TTGQCHITSQNHGYAVD~TSTLPSEFIEYFVNLNDGSNEG1MMHKTRPIFSTQFHPEAKG
NCU07732.1	TTGQCHITSQNHGYAVD~ISTLPSDFKEYFVNLNDGSNEG~MMHKTRPIFSTQFHPEAKG
FG09554.1 	TTGLCHITSQNHGYAID~ASTLPSDFKEYFVNLNDGSNEG~MLHKTRPIFSTQFHPEAKG
AN2243.1  	----------------D1ASTLPSDWKPYFVNLNDSSNEG~MIHKSRPIFSTQFHPEAKG
          	                *  *****::  *******.**** *:**:**************

MG01743.1 	GPMDSSFLFDKYLANVKMFKNSEKVFRDNR---PSQLLLDILSKERVGVEPTP----LAS
NCU07732.1	GPMDSSYLFDKYMENVELFKSNSQVYRDNR---PTQFMIDILSKERVGVEPTP----LSN
FG09554.1 	GPMDSSYLFDKYLQNVQLAKSNQVVFKDNR---PTPLMLDIMSRERVGVEPTP----LAA
AN2243.1  	GPLDSSYLFDIYIDSVKKYKNSQLAFHPSRETIPSPLLVDLLPKERVDVAPTIGMQNVAA
          	**:***:*** *: .*:  *... .:: .*.: *: :::*::.:***.* ** . ..:: 

MG01743.1 	TA--------
NCU07732.1	AA--------
FG09554.1 	SA--------
AN2243.1  	AAAAAAAATA
          	:*::::::::
```
